# Supplementary material for: TTLL1 and TTLL4 polyglutamylases are required for the neurodegenerative phenotypes in pcd mice
Source: PLoS Genet. 2022 Apr 11;18(4):e1010144. doi: 10.1371/journal.pgen.1010144 (PMC9022812; doi:10.1371/journal.pgen.1010144)
Supplement: S1 Table — (DOCX) [file pgen.1010144.s001.docx]

**Table S1.** Primers used for genotyping the knock-out alleles of TTLL

| **Strains** | **Primers** | | **Size of amplicons** |
| --- | --- | --- | --- |
| TTLL1 | WT | 5’-TGACGTTCCCGAGTGTAAGGA-3’ | 410 bp |
|  |  | 5’-GGTGAATACACTGTCACCGTC-3’ |  |
|  | KO | 5’-TTCGACCACCAAGCGAAACATC-3’ | ~800 bp |
|  |  | 5’-GGTGAATACACTGTCACCGTC-3’ |  |
| TTLL4 | WT | 5’-CTTTGCCAGGGTAGGTGGTA-3’ | 671bp |
|  |  | 5’-TACATCCCCGTGTGAACTGA-3’ |  |
|  | KO | 5’-TACCACCTACCCTGGCAAAG-3’ | 880 bp |
|  |  | 5’-GCCATCACGAGATTTCGATT-3’ |  |
| TTLL5 | WT | 5’-CCAAGGTACCCAATGTCCTCT-3’ | 690 bp |
|  |  | 5’-CCAGGTCTCAGCCTTAGTTGT-3’ |  |
|  | KO | 5’-GCTTGCCGAATATCATGGTGG-3’ | 590 bp |
|  |  | 5’-CCAGGTCTCAGCCTTAGTTGT-3’ |  |
| TTLL7 | WT | 5’-GAATTCACTGGCACTCACTCAAATG-3’ | 459 bp |
|  |  | 5’-AAACACAGCACTTGCAGAGGGTT-3’ |  |
|  | KO | 5’-CCGGTCTTGTCGATCAGGATG-3’ | 680 bp |
|  |  | 5’-AAACACAGCACTTGCAGAGGGTT-3’ |  |
| TTLL11 | WT | 5’-CCACCCTGAGTTTTAGCCTTG-3’ | 722 bp |
|  |  | 5’-CCTCTGATGATGGTTGCCACT-3’ |  |
|  | KO | 5’-CCTGCTCTTACTGAAGGCTC-3’ | 570 bp |
|  |  | 5’-CCTCTGATGATGGTTGCCACT-3’ |  |
