# Supplementary material for: TTLL1 and TTLL4 polyglutamylases are required for the neurodegenerative phenotypes in pcd mice
Source: PLoS Genet. 2022 Apr 11;18(4):e1010144. doi: 10.1371/journal.pgen.1010144 (PMC9022812; doi:10.1371/journal.pgen.1010144)
Supplement: S2 Table — (DOCX) [file pgen.1010144.s002.docx]

**Table S2.** Primers used in RT-PCR to determine the transcripts of TTLL

| Gene | Primers |
| --- | --- |
| TTLL1 | 5’-AAGGGTGAAGTGGGTCACTG-3’ |
|  | 5’-TCCGGAACTCTTCCACAAAC-3’ |
| TTLL4 | 5’-TATTTTGGGACTCGGGATGA-3’ |
|  | 5’-CTGGGGCAGGATAAAGGACT-3’ |
| TTLL5 | 5’-GAAGCCATGGTTGTTGGAAG-3’ |
|  | 5’-AACCCTCCTCTCCGATCATT-3’ |
| TTLL7 | 5’-CTTCTAAGCTCTGAACCGCG-3’ |
|  | 5’-TTTCAAACTTTGTTCCGGCAA-3’ |
| TTLL11 | 5’-CTAACCCCCAGAATCTGCAC-3’ |
|  | 5’-CTGGGGTTTGCATTCACTTC-3’ |
