# Supplementary figures and images for: TTLL1 and TTLL4 polyglutamylases are required for the neurodegenerative phenotypes in pcd mice
Source: PLoS Genet. 2022 Apr 11;18(4):e1010144. doi: 10.1371/journal.pgen.1010144 (PMC9022812; doi:10.1371/journal.pgen.1010144)

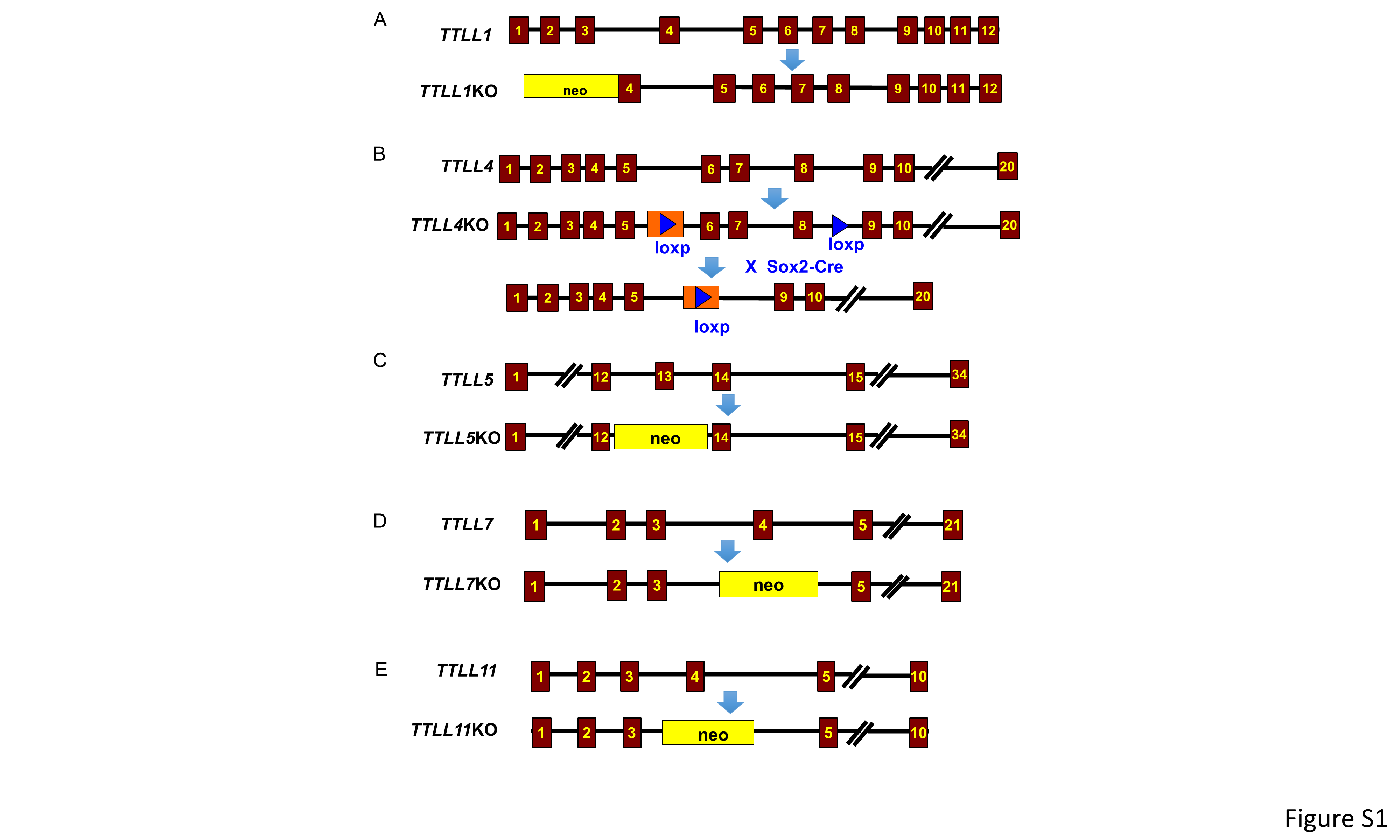

Supplement: S1 Fig — The regions encoding the TTL domain in Ttll1 (A), Ttll5 (C), Ttll7 (D), and Ttll11 (E) were replaced with a neomycin (neo) selection cassette. (B) A conditional knock-out allele of Ttll4 where exons 6–8 were flanked with loxp sites was crossed with the maternally expressing Sox2-cre transgenic mice to create Ttll4KO mice with constitutive deletion of exons 6–8. (TIF) [file pgen.1010144.s004.TIF]

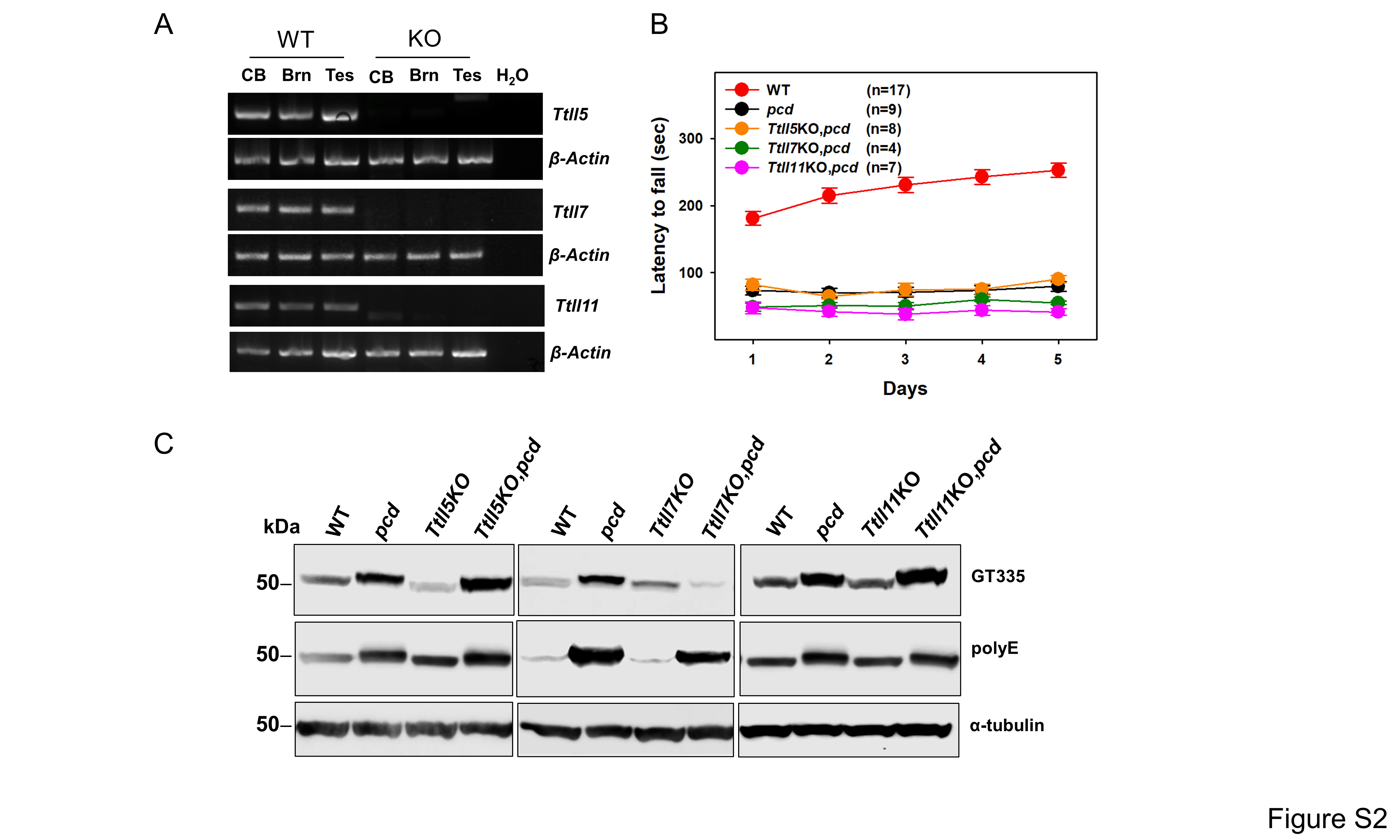

Supplement: S2 Fig — (A) RT-PCR using primers targeting deleted region in Ttll5, 7 or 11KO allele confirmed the absence of Ttll5, 7, or 11 transcripts in cerebellum, brain, and testis of respective KO mice. (B) Rota-rod test of 2-month-old gender-balanced littermates of wild-type; pcd; Ttll5KO,pcd;, Ttll7KO,pcd;, and Ttll11KO,pcd (n = 4–17/genotype) revealed that loss of function of these genes did not improve the locomotor deficit in pcd mice. (C) Tubulin polyglutamylation levels in cerebellar lysates from wild-type (WT), pcd; Ttll5KO; Ttll5KO,pcd; Ttll7KO; Ttll7KO,pcd; Ttll11KO and Ttll11KO,pcd mice. Loss of function of Ttll5 and Ttll11 had little effect on polyglutamylation whereas, loss of function of Ttll7 in pcd markedly reduced GT335 signal, although it did not improve locomotor score. (TIF) [file pgen.1010144.s005.TIF]

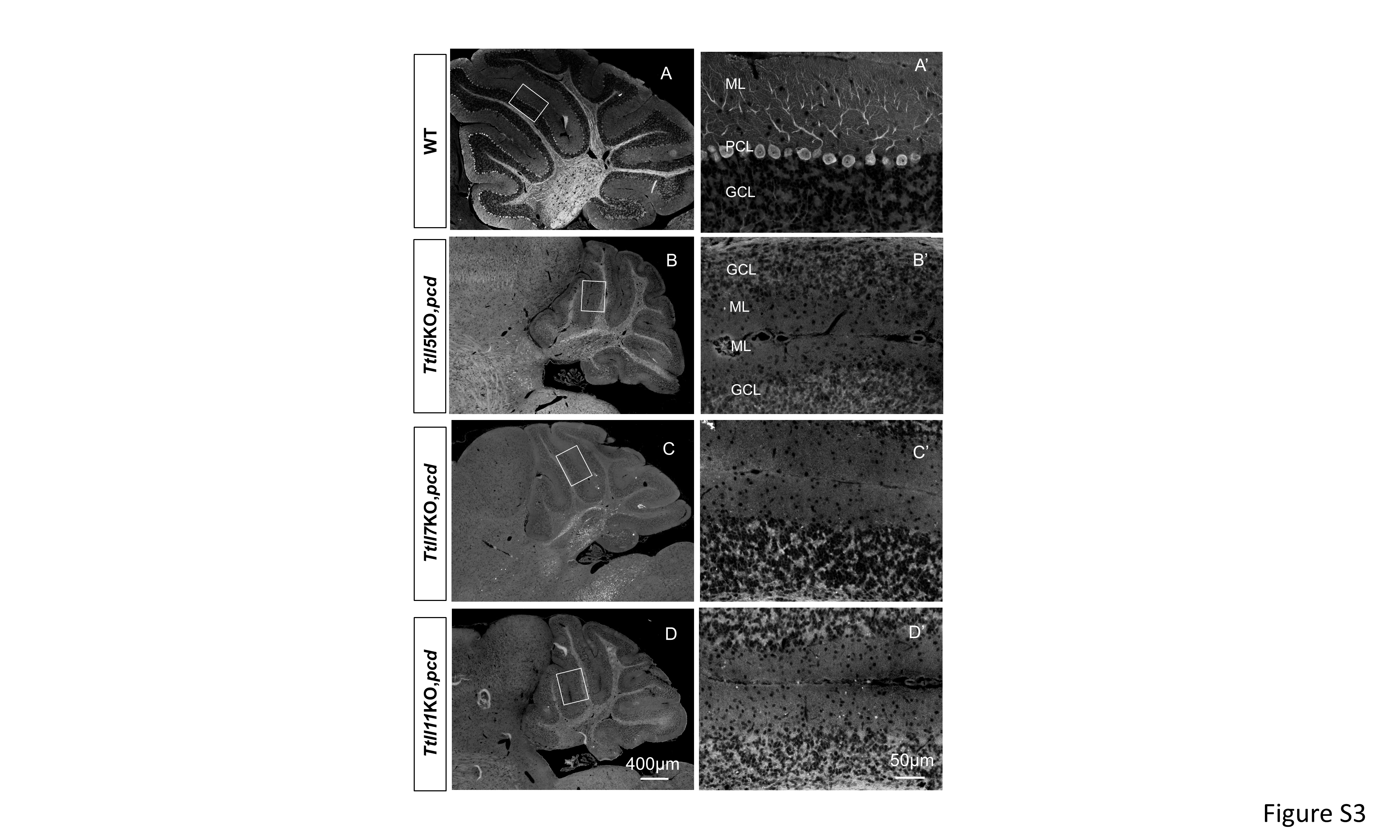

Supplement: S3 Fig — (A-D) Calbindin D-28K immunofluorescence staining of cerebellar sections from 5-month-old wild-type (A and A’), Ttll5KO,pcd (B and B’), Ttll7KO,pcd (C and C’) and Ttll11KO,pcd (D and D’) mice. Note the cerebellum of all TtllKO,pcd mice strains (B-D) is smaller than that of wild-type (A). (A’-D’) Higher magnification of boxed areas in A-D, respectively showed that calbindin-positive Purkinje neurons are not restored in Ttll5KO,pcd, Ttll7KO,pcd or Ttll11KO,pcd mice. ML: Molecular Layer; PCL: Purkinje Cell Layer; GCL: Granule Cell Layer. (TIF) [file pgen.1010144.s006.TIF]

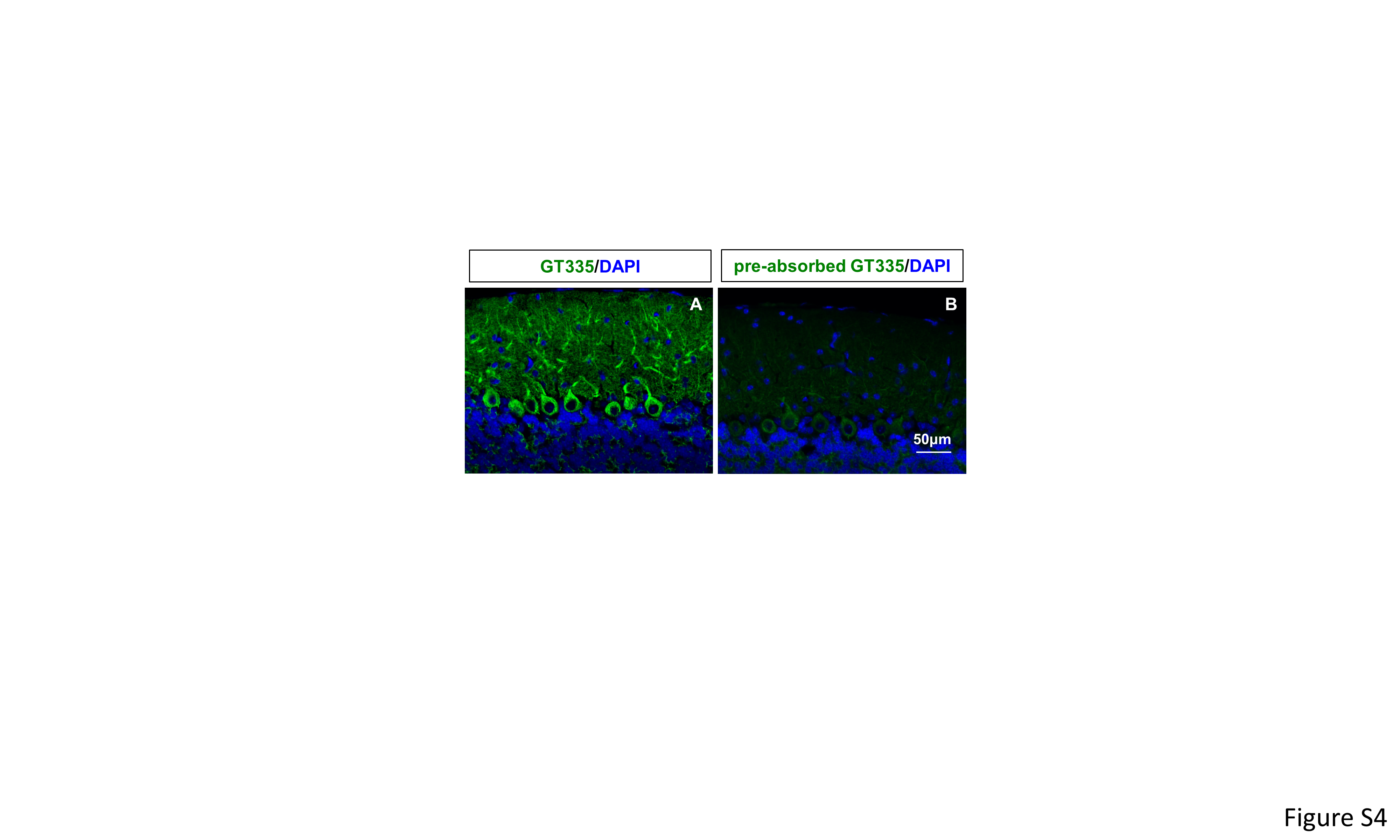

Supplement: S4 Fig — Sections of adult cerebellum were immunostained with GT335 antibody (green) without (A) or with (B) porcine tubulin pre-absorption and nuclei visualized with DAPI (blue). Note large reduction in immunoreactive signal with the pre-absorbed antibody. (TIF) [file pgen.1010144.s007.TIF]

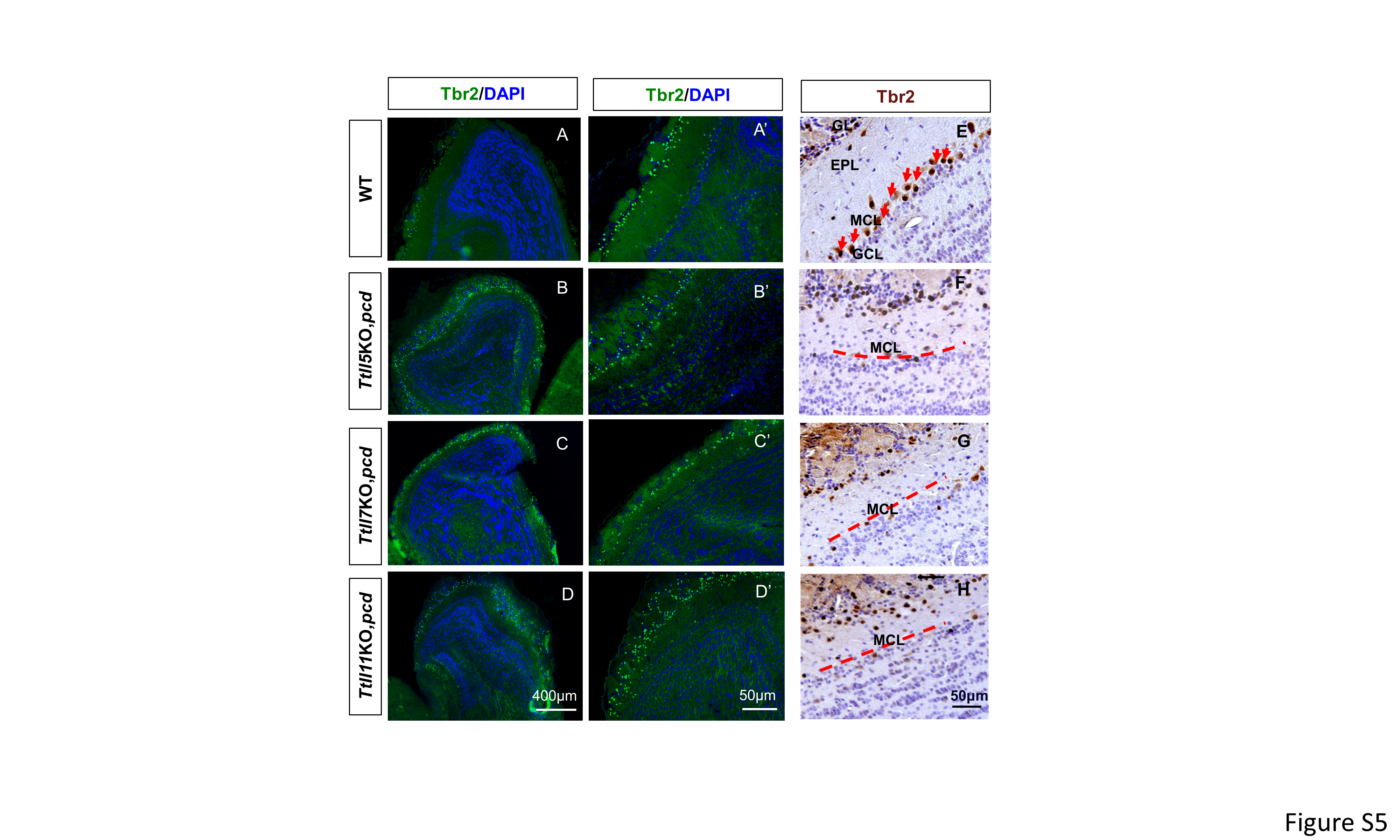

Supplement: S5 Fig — Sections of olfactory bulbs from 5-month-old wild-type (A, A’, E), Ttll5KO,pcd (B, B’, and F), Ttll7KO,pcd (C, C’, and G), and Ttll11KO,pcd (D, D’, and H) mice were immunostained for Tbr2, which recognizes mitral cells and tufted cells located in 2 distinct layers. In all genotypes, Tbr2-positive cells are present in the outer layer where tufted cells are located (A’-D’), whereas Tbr2-positive mitral cells are present in wild-type (A’), but almost completely absent in Ttll5KO,pcd (B’), Ttll7KO,pcd (C’), and Ttll11KO,pcd (D’) mice. (E-H) Bright-field immunohistochemistry images showed that Tbr2-positive mitral cells are present in wild-type mice (E), but largely absent in Ttll5KO,pcd (F), Ttll7KO,pcd (G), or Ttll11KO,pcd (H) double mutants. Arrows indicate Tbr2-positive cells in the MCL and dotted red lines indicate position of MCL. GL: Glomerular Layer; EPL: External Plexiform Layer; MCL: Mitral Cell Layer; GCL: Granule Cell Layer. (TIF) [file pgen.1010144.s008.TIF]
