# Supplementary material for: TTLL1 and TTLL4 polyglutamylases are required for the neurodegenerative phenotypes in pcd mice
Source: PLoS Genet. 2022 Apr 11;18(4):e1010144. doi: 10.1371/journal.pgen.1010144 (PMC9022812; doi:10.1371/journal.pgen.1010144)
Supplement: Attachment — Submitted filename: TTLL Revision Response to Comments.docx [file pgen.1010144.s009.docx]

# We thank the Editor for providing more time to complete revisions which required breeding up mouse strains depleted during the pandemic. Based on reviewers’ feedback we have restructured the entire paper to improve its readability and included new and additional data and Figures as requested. We feel this has greatly strengthened the paper.

# During the period of the revision one paper has appeared in the September edition of EMBO Journal from the Janke/Magiera labs that is relevant to our study. The authors compare loss of TTLL1 and TTLL7 function in pcd mice and essentially come to the same conclusions as us that loss of TTLL7 does not rescue Purkinje cell degeneration yet reduces polyglutamylation in cerebellum of pcd mice. We now cite this study in our revision.

# *Distinct roles of alpha- and beta-tubulin polyglutamylation in controlling axonal transport and in neurodegeneration. Satish Bodakuntia, Xidi Yuan, Mariya Genova, Sudarshan Gadadhar, Sophie Leboucher, Marie-Christine Birling, Dennis Klein, Rudolf Martin, Carsten Janke, Maria M. Magiera. EMBO J. (2021) 40: PMID 34309047*

We thank the reviewers for their constructive comments. With hindsight the reviewers’ general comment about readability of the manuscript is well taken. In response we have restructured the revision to emphasize the unique findings with TTLL1 and TTLL4 KO strains and summarized the findings with the other TTLL mutants in the text and moved much of that data to Supplemental. In addition, we have removed the microarray data as previously presented but extracted quantitative data from that analysis as an orthogonal method to compare Purkinje cell rescue by TTLL1 KO and TTLL4 KO in *pcd* mice. We have also performed additional studies to increase n values, obtain new images and new data.

**For Reviewer 1#**

Major concerns:

1. The authors show that loss of TTLL6 and 11 does not impact Purkinje cell degeneration in pcd mice. However, these genes seem to be barely expressed in Purkinje cells (based on the Allen brain atlas and for TTLL6, the authors’ qPCR data) and thus the inability to rescue this pcd phenotype is likely reflective simply of the spatial or cell type differences in expression rather than functional differences, such as differences in substrate specificity. It is possible that these genes may be able to functionally compensate/act like TTLL1 if they were actually expressed in these cells. Similarly, the differential modulation of the olfactory bulb and retinal phenotype may also simply be reflective of the expression level in these cell types rather than differential substrate specificity. The authors should modify their discussion and results section to clarify this, as it may mislead readers.

We agree with the thrust of the reviewer’s comment and tried to enunciate this in the original manuscript. The situation is indeed very complicated as there are potentially many substrates that may vary between cell type and developmental stage. Beyond that, polyglutamylation homeostasis itself is a complex balance between glutamate addition and removal by enzymes that may have variable expression and activity in different cells and may themselves be influenced by glutamylation as well as other post-translational modifications such as glycylation of substrates that tends to “compete” with glutamylation. We have now restructured the revised manuscript to focus on TTLL1 and TTLL4 as their elimination clearly influence the *pcd* phenotype. We have eliminated TTLL6 from the analysis based on lack of expression in brain. We have now included substantial text in Results and Discussion to highlight the complexity of polyglutamylation homeostasis in vivo and the assumptions being made in interpreting data.

An interesting experiment, though probably beyond the scope of this study, is to investigate whether transgenic overexpression of TTLL6/11 in Purkinje cells can modulate the phenotype of the pcd/TTLL1 double mutants.

This is indeed an interesting suggestion that we had thought about. We have previously published studies showing that while targeted transgenic expression of Nna1/CCP1 to Purkinje cells rescues their degeneration in pcd mice (proving the cell intrinsic role of Nna1 in degeneration in pcd mice), over-expression of two related CCP deglutamylases fail to rescue, implying substrate specificity. A similar strategy might be feasible for TTLLs, but this would require pursuing a transgenic rescue in a pcd/TTLL1/4 null background which given the associated male infertility issues with these strains would be a significant and time-consuming undertaking and as the reviewer notes is beyond the scope of this study.

2. As Purkinje cells are a small fraction of the cells in the cerebellum (about 0.3%), qPCR or western blots on whole cerebellar lysate (consisting mostly of granule cells) do not accurately represent molecular changes in these cells. This makes it difficult to correlate molecular changes to phenotypes, for instance the western blots of glutamylation levels in pcd/ttll4 double mutants to the rescue of Purkinje cell loss. The inclusion of immunohistochemistry or in situ hybridization (e.g. RNAScope) for the different members of this family would be helpful for the interpretation of such results. In addition, (semi)-quantitative immunohistochemistry for tubulin glutamylation in the cerebellum would be helpful to determine the role of tubulin polyglutamylation in the neurodegenerative phenotype.

We agree, this is a fair comment that is common to a number of studies on glutamylation in the context of measurements of bulk extracts of whole brain tissue. We have therefore performed additional sets of immunofluorescence experiments using the GT335 antibody on sections from P19 (prior to frank Purkinje cell degeneration) and adult (post Purkinje cell degeneration) cerebellum (new Fig 6). Our interpretation of these data is that the majority of increased (alpha) tubulin polyglutamylation seen on WB in pcd mice is attributed to granule cells and this is unaffected by loss of TTLL4. While Purkinje cells are prominently stained by GT335 in P19 wild type and pcd cerebellum we do not see overt differences that are influenced by loss of TTLL4. This is also true of Purkinje cells in wild type and Ttll4KO/*pcd* at older ages.

As noted above, polyglutamylation is extremely complex and the field lacks a number of tools that would permit its analysis at the cellular and sub-cellular levels. We have tried to begin a genetic dissection of this in vivo by our systematic genetic rescue strategy in pcd mice. This pointed to TTLL1 and TTLL4 as having a role in degeneration caused by deficiency of the catabolic CCP1 enzyme. We also highlight the complexity by reporting that loss of function of anabolic TTLL5 synergizes with loss of CCP1 to promote degeneration in retina.

3. The inclusion of microarray data from a timepoint at which most Purkinje cells have already been lost in the pcd mouse adds little meaningful data beyond that shown by the histology as gene expression changes are reflective of the absence of these cells.

We did not articulate adequately the reason for including this data. To simplify the paper, we have removed the holistic microarray data as previously presented. However, we have extracted quantitative data from that analysis as an orthogonal method to compare the degree of Purkinje cell rescue by *Ttll1*KO and *Ttll4*KO in *pcd* mice by expression of three Purkinje cell marker genes. We feel this also helps address Reviewer #2 concerns about partial rescue of *pcd* cerebellar phenotype by TTLL4 loss. Unlike in retina, there is no statistically significant differences between marker gene expression in cerebellum of WT, *Ttll1*KO/*pcd* and *Ttll4*KO/*pcd* mice whereas there is a dramatic reduction in *pcd*. These data are presented in new figure 4.

Minor suggestions:

1. It is unclear which lobule is shown in the cerebellar histology, or even if the same lobule is shown for the different genotypes (Fig. 2C, 3D etc). This should be clarified, and if possible, a low magnification image of the entire cerebellum should be included.

As there were concerns from both reviewers concerning presentation of images we have repeated most experiments using immunofluorescence to even out issues with color balance and also include low power magnifications of whole cerebellum. We feel this strengthens the paper and aligns the methodology with other publications in the field of polyglutamylation in *pcd*. Additionally, as there were concerns about partial rescue by TTLL4 loss in cerebellum, we also include images from a 10-month-old animal showing no obvious Purkinje cell loss across a broad swath of the cerebellum in *Ttll4*KO/*pcd* mice.

2. Quantification/statistical analysis should be included for the western blots. In addition, some of the western blots including Fig. 3B and 4B have saturated signal/shadow bands.

This is a relevant request. We have now redone a series of the western blots in multiple (>3) animals of each genotype and quantified expression and incorporated this data in graphical form into a new Figure 5. Note that we now only focus on TTLL1 and TTLL4, so other data is relegated to Supplemental. The take home message is that TTLL1 loss reduces glutamylation in *pcd* mice whereas glutamylation in *Ttll4*KO/*pcd* mice is not statistically different from the elevated levels in *pcd* mice.

3. Do TTLL6/11 affect testes function/male infertility?

In our hands both TTLL6KO and TTLL11KO male mice are fertile.

4. The authors mention that loss of function mutations in TTLL5 have been shown to cause photoreceptor loss in mice (line 485). Has photoreceptor loss indeed been observed in mouse models? If so, can the authors discuss the discrepancy with their data?

I am not sure there is a discrepancy, but we might have been more explicit in the text. In the study of Sun et al., (2016) they report photoreceptor loss in old (20-22-months-old) TTLL5-null mice. This is said to be a model for retina disease-causing TTLL5 mutant alleles in humans that are apparent at ~40 years of age. As we were interested in the interaction of TTLLs with loss of Nna1/CCP1 phenotypes in *pcd* mice, we never extended our retina studies past 5-months of age. At 5 months there is no discernible loss of photoreceptors in TTLL5KO mice. However, we did observe that in *Ttll5*KO/*pcd* retina, photoreceptor loss (~90% loss) was much more severe than in *pcd* alone (~70% loss). We have modified the figure (new figure 9) and text to emphasize our data and clarify the “discrepancy” with the literature. As noted above this is an example of where loss of an anabolic enzyme (TTLL5) and a catabolic enzyme (Nna1/CCP1) act synergistically in photoreceptor degeneration.

**For Reviewer 2#**

Major points:

1) Fig. 1A: First of all, why did the authors omit TTLL2, TTLL9 and TTLL13 which have been predicted / demonstrated to be glutamylases?

See Below: Expression of all three TTLLs are low (TTLL9) or undetectable (TTLL2 and TTLL13) in brain (all are high in testis) (new Fig 1) and all appear to be absent in mitral cells and only a trace of TTLL9 signal is evident in Purkinje cells according to the Allen Atlas. Furthermore, we do not yet have access to a TTLL9-null mouse model. Hence our focus on TTLL1, 4, 5, 7 and 11.

However, the main problem with this figure and the conclusions drawn from it is the fact that it is very difficult to conclude the roles of these enzymes in the tested tissues from these analyses. How can Q-PCR data from different genes be compared?

See below: We agree there are variables that can affect quantification in qRT-PCR. That said, we use a version of qRT-PCR that incorporates standard curves for every TTLL gene and provides the ability to calculate transcript copy number for each gene. We provide a reference for the method. This does at a first approximation permit comparison of abundance of one gene versus another, particularly where differences are large. The reviewer alludes to papers that report “abundance” of TTLLs but these also rely on similar approaches. As it’s not a critical point to this study, we have modified text appropriately to deemphasize this.

Most likely not at all. What can, however, be compared is the relative expression of the same gene in different organs or tissues. For TTLL6, for instance, it is obvious that it might not play a role in the nervous system, as its expression levels are very low compared to testes. All other enzymes show similar expression levels in brain and testes. To make a convincing point about the specific expression of the enzymes in the nervous system, the authors must compare their expression levels in the brain to the expression levels in other organs, and preferably organs where polyglutamylation plays a less important role (testes are surely not the best control here). Without this comparison, it is hard to tell if the numbers the authors obtained stand for a strong or weak expression.

See below: Agree, new Figure 1 contains data on liver and kidney in addition to testis and brain regions.

Finally, it is not clear how many times these analyses were repeated (no statistical analysis of the data). Also, representing the Q-PCR data in the form of diagrams would improve the readability of the figure.

See below: New Figure 1 includes full statistical analysis.

We appreciate the reviewer’s inter-related comments and have completely repeated the qRT-PCR experiment to include multiple brain regions, testis and several organs (liver and kidney) not thought to be impaired in pcd mice. This is now part of a new Figure 1 that also includes a more didactic view of polyglutamylation as also suggested by reviewer.

This provides a general picture of abundance of each TTLL in different tissues which the reviewer requested. Besides the testis being the site of highest expression of TTLLs (a well-established fact), the data also show that TTLL levels in brain are generally higher than in liver and kidney. The data also show that several polyglutamylase TTLLs are not expressed or expressed at low levels in brain and hence we did not investigate the effect of their elimination on *pcd* phenotypes. For completeness, we also measured expression on non-glutamylase TTLLs and note that we also did not pursue them but have chosen to focus on TTLL1 and TTLL4 in the main text to aid readability.

2) Quality of data representation: The paper has some major problems with the quality and the readability of the figures. This is not only a problem for the readability of the manuscript, it might also affect the interpretation of the results. The two main concerns are:

2a) Immunoblots lack molecular weight markers and are very closely cropped. This is not good practice.

We agree and have added molecular weight markers. The new figure 5 is less tightly cropped.

2b) The authors never comment whether they can distinguish alpha- and beta-tubulin in their blots, which would be helpful as they interpret a lot of their results in the light of the enzymatic specificities of the enzymes.

We agree and state that the specificities of TTLLs have largely been characterized *in vitro* and provide this information to the reader to aid in following the paper. We do see immune-reactive bands on western blot consistent with alpha- and beta-tubulin as reported in the literature. However, we have not purified them and independently confirmed their identity by mass spectrometry or another orthogonal method. Our data indicate that TTLL1KO reduces polyglutamylation signal in general whereas TTLL4 loss has no statistically significant effect. Of course, it’s possible that there are small changes in glutamylation that are critical and beyond resolution in such bulk assays, or perhaps only a minor subtype of tubulin is key to the phenomena. It also leaves open the possibility that tubulins are not the critical substrate for TTLL4. We did enunciate these various possibilities in the original manuscript but have reworked it to emphasize this. We have also performed IF using the GT335 antibody and conclude that the increased alpha-tubulin seen on western blot in *pcd* cerebellum appears to emanate from granule cells and this is not affected by loss of TTLL4 new figure 6.

2c) All polyE blots in this manuscript appear to be overloaded, and can therefore not reveal subtle differences in polyglutamylation levels that might occur in some cases. While the previously reported very strong difference between wild type and CCP1-KO (pcd) is visible under these experimental conditions, the authors might have missed a more subtle decrease in polyE signal, for instance in CCP1/TTLL4 dKO mice that show partial Purkinje-cell survival. Being able to see such subtle differences would completely change the interpretation of the paper.

We agree some images of polyE look overloaded or over-exposed and have taken steps to improve visual presentation. Furthermore, as we now only focus on TTLL1 and TTLL4 in the manuscript we have performed additional experiments (including loading varying amounts of protein) and quantitated the signals. The latter is presented in graphical form within the new figure 5.

2d) Histology images are not homogenously represented. First, they are so different in terms of colour, and region selected it is not obvious for a reader that the brain regions depicted are comparable. This concerns Fig. 2C, 3D, 4C and 6 (for instance, compare panels in Fig. 2C – they all look completely different). Images need to be better processed, comparable regions should be shown. Brain layers should be labelled. Moreover, given that brain histology is basically the key readout of this work, entire cerebella should be shown additionally to the zoomed-in region.

Additional mice have been analyzed using IF and new figures (3, 4, 7, 8 and 9) have been generated. These include low power annotated images to provide anatomical perspective as requested as well as information on where higher power images were acquired.

3) It is not clear why the authors first analyse the TTLL6-KO mice, having shown in Fig. 1A that this enzyme is not expressed in the nervous system? This is counter-intuitive, and becomes particularly perturbing at line 202, where the authors simply conclude that TTLL1, TTLL6 and TTLL11 elongate polyglutamylation on alpha-tubulin, but only TTLL1 appears to contribute to the degeneration of Purkinje cells – citing Fig. 1A. Going to Fig. 1A now, the reader discovers that TTLL6 is not expressed in cerebellum (making the lack of rescue obvious), but TTLL11 is Q-PCR-wise more than 2x more expressed than TTLL1. So how can this be explained? This most likely needs to be put in the context of what Q-PCR data really tell us (see point 1) above).

We agree and have removed TTLL6 data from the analysis based on reviewer’s comment that it is not expressed. Furthermore, we now only include full data for TTLL1 and TTLL4 in main text and have moved the majority of data for other TTLLs to supplementary as it may be useful to other investigators who might want to assess their role in *pcd* mice. Bottom line here is that we have taken a genetic approach to dissect which TTLLs are implicated in neurodegeneration caused by a deficiency of Nna1/CCP1. We are not trying to explain why other TTLLs don’t have an effect.

4) The most striking discovery of this paper is the (partial) rescue the CCP1-related phenotypes by knockout of TTLL4. However, it raises the important question of the underlying mechanism given that in the western blots it seems that TTLL4-KO does not reduce hyperglutamylation, while TTLL1-KO does. As mentioned in point 2c), there might be a slight reduction of polyglutamylation the authors have not detected because of the way they perform their blots, but the question still remains of how TTLL4-KO can rescue given that is does not lower polyglutamylation as does TTLL1-KO. One possible answer could be that in fact all of the observed rescue phenotypes are partial.

We have now performed WB on additional mice and quantified the data and there is no statistically significant difference between *pcd* and *Ttll4*KO/*pcd* polyglutamylation levels (new figure 5). We concur that in bulk assays it is always possible that there is a small change that cannot be detected, but this is true for all such assays in the literature. To try to address this possibility, we have added a new Figure 6 looking at GT335 immunoreactivity in sections from cerebellum of P19 (pre-degeneration) and adult (post-degeneration) wild type, *pcd* and *Ttll4*KO/*pcd* mice. We do observe changes in granule cell immunofluorescence in P19 *pcd* mice compared to wild type but this is also evident in *Ttll4*KO/*pcd* cerebellum. We do not see obvious differences in staining Purkinje cells (which in our hands have prominent GT335-immunoreactivity). So in cerebellum we believe the increase (alpha)-tubulin in *pcd* comes at least in part from granule cells and is not dependent on TTLL4 status. In hindsight this was pretty obvious as on western blots of *pcd* adult mice there are few or no Purkinje cells yet the increased GT335 band is still evident.

The reviewer asks how TTLL4 works if it doesn’t change tubulin hyperglutamylation. We suggested a range of possibilities up to an including that tubulin is not the relevant substrate. We have reiterated this in the revised manuscript and also point to the limitations imposed by the reagents and tools available to investigate polyglutamylation homeostasis in single cells *in vivo*.

To address the question of whether TTLL4 loss provides partial protection we have also performed assessment of Purkinje cell and mitral cells survival in older mice and performed orthogonal assays aimed at measuring expression of genes that are specifically expressed in Purkinje cells. This new data is presented in modified figures 4 and 8 and clearly demonstrate substantial survival of both cell types at 10 months of age in *Ttll4KO*/*pcd* mice. It also shows that TTLL1- and TTLL4 deficiency support Purkinje cell survival to equivalent levels on a *pcd* background. These new data are incorporated into a new figure 4. Of course, it is possible that cell death is still occurring a slow rate but given the time constraints of the review we could not extend further. Nevertheless, it is obvious that from a genetic perspective TTLL4 loss attenuates death of Purkinje and mitral cells.

Indeed, the authors show that TTLL4-KO can only partially rescue the CCP1-induced degeneration of the retina, but they claim at the same time that it can fully rescue mitral and Purkinje cells based on snapshots of olfactory bulb and cerebellum without any quantification or multiple experiments. Given that in mouse models in which Purkinje cells degenerate only partially, this partial degermation is not homogenous throughout the cerebellum (e.g. Fig. 1G in Liu Y, Lee JW, Ackerman SL (2015) Mutations in the Microtubule-Associated Protein 1A (Map1a) Gene Cause Purkinje Cell Degeneration. J Neurosci 35: 4587-4598), it would be first of all essential to show images of the entire cerebellum (and similarly, the entire olfactory bulb) in order to show that the region the authors have presented currently is representative. Moreover, the authors might attempt to quantify the number of Purkinje cells (and mitral cells) in the different mouse models to determine whether there is a partial degeneration similar to what they see in the retina. Finally, it might be worth checking whether the protective effect of TTLL4 is as efficient as the one by TTLL1 by investigating older mice.

We agree that degeneration of Purkinje cells in *pcd* and other mutants can have temporo-spatial differences. We now include low power images in a new Figure 4 and we have examined older mice (within the temporal constraints of the review) as suggested. We also now discuss this more explicitly in the Discussion section.

As the olfactory bulb shrinks in pcd, and as multiple Tbr2-positive cell classes are present (some in the same general region as mitral cells) we have not made calculations of how many mitral cells survive in Ttll1KO/pcd and Ttll4KO/pcd mice. Unfortunately, we do not have definitive markers for mitral cells so we cannot perform comparative studies of TTLL1 and TTLL4 dependence.

I am not sure we ever used the phrase “fully rescue” that would certainly be an overstatement on our part. We have tried to be more cautious in the text and have avoided the term rescue.

5) The gene expression study in Fig. 5 is awkward: all the genes that the authors find to be downregulated are Purkinje-cell specific genes. So obviously, in all mouse models where the Purkinje cells are lost, the genes specifically expressed in these cells cannot be detected anymore. But this does by no means prove their downregulation, but simply mirrors the loss of Purkinje cells..

We did not articulate adequately the reason for including this data. To simplify the paper, we have removed the holistic microarray data as previously presented. However, we have extracted quantitative data from that analysis as an orthogonal method to compare the degree of Purkinje cell rescue by loss of TTLL1 or TTLL4 in *pcd* mice via expression of three Purkinje cell marker genes. We feel this also helps address the concerns about partial rescue of *pcd* cerebellar phenotype by TTLL4 loss. Unlike in retina, there is no statistically significant differences between marker gene expression in cerebellum of WT, *Ttll1*KO/*pcd* and *Ttll4*KO/*pcd* mice whereas there is a dramatic reduction in *pcd*. These data are included in a new Figure 4.

Minor points:

1) In the abstract, the authors write “Genetic disruption of polyglutamylation causes neurodegenerative phenotype”. This is misleading, as it could be interpreted as loss of glutamylation (“disruption”). However, what really happens is that accumulating polyglutamylation leads to degeneration. This should be re-formulated.

We have changed the abstract as suggested by reviewer as we agree with it in the context of cerebellar Pj cell degeneration. In fact, we had already stated this in the body of the paper. However, reduced polyglutamylation also causes degeneration in the eye in the context of loss of function of TTLL5 and in testis in the context of TTLL1 deficiency, so indeed genetic disruption of polyglutamylation does lead to degenerative phenotypes.

2) The authors omit the notion that while all glutamylases have a previously demonstrated preference for initiation or elongation, as well as for alpha- or beta-tubulin, these are preferences and not exclusive activities. In other words, one cannot exclude that TTLL1 initiates the glutamate chains it later elongates. They should consider the potential promiscuity of the enzymes when discussing their findings.

Agree, and believe we had said something along these lines in the original manuscript. We have completely revised sections of the paper dealing with this subject and cited a recent paper showing TTLL1 can act as an beta-tubulin elongator.

3) Fig. 1B: it is important to introduce the reader to polyglutamylation and the involved enzymes. However, this could be done a bit more intuitive than in the current figure that oversimplifies a lot. Why not introducing the notion of alpha- and beta-tubulin preferences into this schematic, and remove it from Fig. 1A, where data are shown?

We have generated a new figure 1 showing the process more didactically and placed it at start of Results and introduced the background of polyglutamylation in the text at the same point.

4) line 182: the authors must explain the connection between PGs1 and TTLL1 mutant mice.

This is now explained in Discussion. Our TTLL1 knockout is null for the catalytic subunit of a multi-protein complex that represent the holoenzyme. PGs1 is another subunit of the complex and its knocking-out phenocopies our TTLL1 KO (actually our mutant may be slightly more severe based on the testicular phenotype).

5) in Fig. 7, the zoom does not really reveal what the authors want to show. A more explicit representation should be sought.

Figure has been redone as requested. Figure 7 is now figure 9.

6) in Fig. 8, single-channel images as in Fig. 7 would be useful to clearly see the phenotype the authors want to point out. Fig. 7 and 8 could be combined.

Figure has been redone and combined (new figure 9).

7) In the discussion, the authors propose that their observation of the importance of TTLL4 for Purkinje cells might be explained by both, alpha- and beta-tubulin glutamylation participating in this phenotype. However, they did not show that TTLL4 is contributing to beta-tubulin glutamylation in neurons or brain tissue, and they also do not discuss why in this case TTLL7, a known beta-tubulin glutamylase with a very strong expression in brain, and important functions in neurons (Ikegami et al., 2006) does not rescue CCP1-mediated degeneration.

While performing this revision, a paper appeared showing the TTLL7KO does reduce polyglutamylation in cerebellum but does not rescue degeneration in *pcd* (the same results we report). We cite this paper. Our limitation is that we do not have access to a reagent that can specifically assess beta-tubulin glutamylation status in Purkinje cells.
