# Supplementary material for: TTLL1 and TTLL4 polyglutamylases are required for the neurodegenerative phenotypes in pcd mice
Source: PLoS Genet. 2022 Apr 11;18(4):e1010144. doi: 10.1371/journal.pgen.1010144 (PMC9022812; doi:10.1371/journal.pgen.1010144)
Supplement: Attachment — Submitted filename: TTLL Revision 1 Response to comments.docx [file pgen.1010144.s010.docx]

We thank both reviewers for their constructive comments that we feel have enhanced the paper.

**Comments to the Authors:**

Reviewer #1: The authors have addressed my concerns, and the manuscript is much improved. I now support its publication. I would suggest that the authors label the relevant genotypes on the panels of their immunofluorescence figures (Fig3,4,6, 7-9) to make things easier for readers, and avoid labels such as panel Aa''.

We agree with this suggestion and have modified all figures to indicate genotypes and changed text appropriately.

Reviewer #2: The authors present a thoroughly revised manuscripts in which they carefully addressed all referees’ comments. As a result, the revised manuscript has hugely improved. Most importantly, the authors have performed a series of new experiments to prove the reproducibility of their observations, which was one of the key concerns in the first round of review. They have further re-focused the story onto the key findings that TTLL1 and TTLL4, but none of the other tested TTLL enzymes play a role in neurodegeneration induced by accumulation of polyglutamylation. This improves the logical flow of the new manuscript, while all important information is still available in the supplementary material.

Given the solidness of this work, it will be a strong contribution to the further mechanistic understanding of the newly emerging role of the tubulin modification polyglutamylation in neurodegeneration. In particular, the observation of the authors that the enzyme TTLL4 contributes to this novel form of degeneration despite the fact that TTLL4 is not the most-strongly expressed TTLL in the brain is intriguing and will help to further elucidate the potentially complex mechanisms leading to the loss of neurons in the future.

Overall, the revised manuscript shows no major weaknesses, experimental data are solid, and results are meticulously discussed. A couple of minor concerns should be addressed before considering the paper for publication:

1) The authors should carefully re-read the manuscript. It appears that there are parts that have not been fully revised, as for instance some figure numbers are wrong: in the discussion they cite Fig 9 for testes, whereas this is Fig 10 in the revised manuscript.

We have carefully reviewed the (re-revised) manuscript and corrected any errors.

2) In the introduction (first sentence) the authors write that polyglutamylation is involved in neurodegeneration. This is a bold statement, and could mislead the reader into thinking of common neurodegenerative disorders such as Alzheimer’s disease. Specifying that polyglutamylation is involved in a rare condition of childhood degeneration would be more appropriate.

The reviewer’s statement is correct for Nna1/Ccp1, however, mutations of additional genes involved in homeostasis of polyglutamylation (eg TTLL5 discussed in this study), also result in neurodegenerative phenotypes. We do agree that these are rare disorders and so adjusted our statement to reflect this.

3) In Fig 4A, panel a, the axon tracts stained with Calbindin are less prominent in wildtype as compared to the mutant in panel b. This is most likely related to the choice of the brain section, but could give the misleading impression that wildtype Purkinje cells have a smaller axon tract as compared to the mutant. The authors might consider replacing the image.

We matched cerebellar sections to show the same lobes to address reviewer’s original concern that rescue of Purkinje cell degeneration in TTLL4/pcd mice was lobe/region specific. This is the best matched section we have from that perspective and would prefer to retain it.

4) What makes the manuscript really difficult to read are the figures. There are a number of style issues with the figures, which the authors might want to address:

• Fig 1B: a table packed with so many numbers is far from being easy to read. Why don’t the authors choose to represent their data as graphs?

We did consider graphical representation of data in Fig 1B. However, the reviewer will appreciate that there are orders of magnitude differences (~1-21,000) between expression of various TTLLs in different tissues, which doesn’t make for easy graphical presentation and would obscure the statistical component that reviewer questioned in original review. Therefore, we feel that this very large dataset is best represented in this tabular format.

• Fig 3, 4A, 6, 7, 8, 9 – without a labelling of the panels (genotypes, antibodies used, etc.) these figures are very hard to decipher (one goes forth and back between figure and legend many times before being sure to correctly understand the data). Given that the authors have shown willingness to put such labels in Fig 10, they might want to consider doing so consistently in all figures.

We concur with this suggestion and have added genotypes.

• In most figures showing immunohistochemistry, the authors wrote text labels over the figure panels. Most of these labels are very small and hard to read, and sometimes it is even impossible to read them because of the image below. Bold letters, and perhaps some background shading, could help here. Scale bars are also very thin lines, thus hard to spot. Given that different magnifications are shown side-by-side, it would not heart to directly label the scale bars in the panels.

We have revised all figures as suggested to make the labelling and annotated scale bars more legible.

• Fig 3, 4A and 6 are monochrome images: why do the authors show them in green? They would be much more contrasted in black and white.

We are agnostic on the relative merits of color vs. black and white. Since reviewer seems to favor black and white we have replaced color images in Figs 3, 4A and 6 with black and white.

• To make Fig 7 and 8 more readable for the non-specialist, a better labelling of the layers, addition of zoom images to show details, plus a schematic representation of the olfactory bulb could be very helpful.

We have modified labelling as requested to make more legible.

• Fig 2C,D – the authors plot 4 different measurements using very hard-to-distinguish symbols. They might consider the use of color to make these graphs easier to read.

Agreed, we now use color coding.

• Fig 4B, 5C,D: the text in these graphs is much too small to be readable.

• Some of the panels showing immunohistochemistry images are very small, in particular Fig 3 and 8. Given the wealth of important information on those images, they could be much larger than for instance an immunoblot with only 4 protein bands in Fig 5.

We have fixed the labelling and enlarged figures as suggested.

**Have all data underlying the figures and results presented in the manuscript been provided?** Large-scale datasets should be made available via a public repository as described in the *PLOS Genetics* [data availability policy](https://nam11.safelinks.protection.outlook.com/?url=http%3A%2F%2Fjournals.plos.org%2Fplosgenetics%2Fs%2Fdata-availability&data=04%7C01%7Cyongqi.rong%40stjude.org%7C0fa69ac4be9b4310003008d9f24b3850%7C22340fa892264871b677d3b3e377af72%7C0%7C0%7C637807227206086680%7CUnknown%7CTWFpbGZsb3d8eyJWIjoiMC4wLjAwMDAiLCJQIjoiV2luMzIiLCJBTiI6Ik1haWwiLCJXVCI6Mn0%3D%7C3000&sdata=H1wtFiOxQhumjzyuJycMSe51oGE7sbXGOlZvNILSLAc%3D&reserved=0), and numerical data that underlies graphs or summary statistics should be provided in spreadsheet form as supporting information.

Reviewer #1: Yes

Reviewer #2: **No:** If I am not mistaken, there are no numerical data to the graphs shown in the manuscript, please verify.

We provided supporting data in spreadsheet form now.

PLOS authors have the option to publish the peer review history of their article ([what does this mean?](https://nam11.safelinks.protection.outlook.com/?url=https%3A%2F%2Fjournals.plos.org%2Fplosgenetics%2Fs%2Feditorial-and-peer-review-process%23loc-peer-review-history&data=04%7C01%7Cyongqi.rong%40stjude.org%7C0fa69ac4be9b4310003008d9f24b3850%7C22340fa892264871b677d3b3e377af72%7C0%7C0%7C637807227206086680%7CUnknown%7CTWFpbGZsb3d8eyJWIjoiMC4wLjAwMDAiLCJQIjoiV2luMzIiLCJBTiI6Ik1haWwiLCJXVCI6Mn0%3D%7C3000&sdata=7DBumgZvEpf4pFkpaDNCfP2gfcQR9EdthhFAe99ajAg%3D&reserved=0)). If published, this will include your full peer review and any attached files.   If you choose “no”, your identity will remain anonymous but your review may still be made public.   **Do you want your identity to be public for this peer review?** For information about this choice, including consent withdrawal, please see our [Privacy Policy](https://nam11.safelinks.protection.outlook.com/?url=https%3A%2F%2Fwww.plos.org%2Fprivacy-policy&data=04%7C01%7Cyongqi.rong%40stjude.org%7C0fa69ac4be9b4310003008d9f24b3850%7C22340fa892264871b677d3b3e377af72%7C0%7C0%7C637807227206086680%7CUnknown%7CTWFpbGZsb3d8eyJWIjoiMC4wLjAwMDAiLCJQIjoiV2luMzIiLCJBTiI6Ik1haWwiLCJXVCI6Mn0%3D%7C3000&sdata=x7QVbvvwLtX8tXu5zW7YeOcTXlAx89IfUWybrG%2FB6%2F0%3D&reserved=0).

Reviewer #1: No

Reviewer #2: No
