# Supplementary material for: TTLL1 and TTLL4 polyglutamylases are required for the neurodegenerative phenotypes in pcd mice
Source: PLoS Genet. 2022 Apr 11;18(4):e1010144. doi: 10.1371/journal.pgen.1010144 (PMC9022812; doi:10.1371/journal.pgen.1010144)
Supplement: Attachment — Submitted filename: Editor comments to the authors.docx [file pgen.1010144.s011.docx]

We thank the editors for your constructive comments.

Editors Comments to Authors

We reviewed your revision and we agree that most of the changes you made are responsive and acceptable.  But in the interest of readability, as the reviewer had been concerned, there are still a few minor changes that we would ask that you make before the manuscript is formally accepted.  Once you address these we will be poised to make the editorial decision very quickly.

First, in principle it is okay if you leave Figure 1b in table form, but it is still an eyeful to look at. Since the gene expression values are whole integers with no decimals, the second decimal place in the SEM is unnecessary and clutters up the table – please use only one decimal place for the SEM values.  Also, please put a space between the expression value and the ± symbol.

We agree with this suggestion and have modified Figure 1B.

Your including genotypes in the figures as the reviewer asked is helpful, but it needs to be clearer still.  It is acceptable to use shorthand gene symbols in the genotypes rather than full formal symbols in the figures, as long as the shorthand is defined somewhere clearly in the text (e.g. Methods).  Thus, pcd should be defined as Agtpbp1 (italicized) pcd (superscript, non italicized, no spaces), and then if you mean homozygous spell that out too (e.g. Agtpbp1 pcd/Agtpbp1 pcd or heterozygous (Agtpbp1 pcd/Agtpbp1 +).  Similar for Ttl1 KO (there is probably an official gene symbol for this KO allele – you can check with JAX if one has been registered; if not, you can use the minus sign to indicate ko, i.e. -).  Also, for pcd specifically in some figures you use pcd and others pcd^3J; please be consistent if you only used one of these.  Last, the double mutant shorthanding is confusing because you are using a slash which is typically reserved for use in genotype copy number; instead of the slash, to indicate double mutants you should use a comma.  That is, if you are going to shorthand Ttl1 mice as Ttl1KO and Agtpbp1 as pcd, and specify in the Methods that these are homozygous, then you could use in the shorthand for the figure: Ttl1KO, pcd).  If there are mixed copy numbers, then that could be further specified, e.g. Ttl1KO/+, pcd/pcd.

We agree with this suggestion and have modified all figures and changed text appropriately.

Again, once these issues are addressed we will finalize a decision rapidly.
